# Comparative Toxicogenomics Database’s 20th anniversary: update 2025

**DOI:** 10.1093/nar/gkae883

**Published:** 2024-10-10

**Authors:** Allan Peter Davis, Thomas C Wiegers, Daniela Sciaky, Fern Barkalow, Melissa Strong, Brent Wyatt, Jolene Wiegers, Roy McMorran, Sakib Abrar, Carolyn J Mattingly

**Affiliations:** Department of Biological Sciences, 3510 Thomas Hall, 112 Derieux Place, North Carolina State University, Raleigh, NC 27695, USA; Department of Biological Sciences, 3510 Thomas Hall, 112 Derieux Place, North Carolina State University, Raleigh, NC 27695, USA; Department of Biological Sciences, 3510 Thomas Hall, 112 Derieux Place, North Carolina State University, Raleigh, NC 27695, USA; Department of Biological Sciences, 3510 Thomas Hall, 112 Derieux Place, North Carolina State University, Raleigh, NC 27695, USA; Department of Biological Sciences, 3510 Thomas Hall, 112 Derieux Place, North Carolina State University, Raleigh, NC 27695, USA; Department of Biological Sciences, 3510 Thomas Hall, 112 Derieux Place, North Carolina State University, Raleigh, NC 27695, USA; Department of Biological Sciences, 3510 Thomas Hall, 112 Derieux Place, North Carolina State University, Raleigh, NC 27695, USA; Department of Biological Sciences, 3510 Thomas Hall, 112 Derieux Place, North Carolina State University, Raleigh, NC 27695, USA; Department of Biological Sciences, 3510 Thomas Hall, 112 Derieux Place, North Carolina State University, Raleigh, NC 27695, USA; Department of Biological Sciences, 3510 Thomas Hall, 112 Derieux Place, North Carolina State University, Raleigh, NC 27695, USA; Center for Human Health and the Environment, 850 Main Campus Drive, North Carolina State University, Raleigh, NC 27695, USA

## Abstract

For 20 years, the Comparative Toxicogenomics Database (CTD; https://ctdbase.org) has provided high-quality, literature-based curated content describing how environmental chemicals affect human health. Today, CTD includes over 94 million toxicogenomic connections relating chemicals, genes/proteins, phenotypes, anatomical terms, diseases, comparative species, pathways and exposures. In this 20th year anniversary update, we reflect on CTD’s remarkable growth and provide an overview of the increased data content and new features, including enhancements to the curation workflow (e.g. new exposure curation tool and expanded use of natural language processing), added functionality (e.g. improvements to CTD Tetramers and Pathway View tools) and significant upgrades to software and infrastructure. Linking lab-based core curation with real-world human exposure curation via the use of controlled vocabularies facilitates analysis of content across the entire environmental health continuum, from molecular toxicological mechanisms to the population level, and vice versa. The ‘prototype database’ originally described in 2004 has evolved into a premier, sophisticated, highly cited and well-engineered knowledgebase and discoverybase that is utilized by scientists worldwide to design testable hypotheses about environmental health.

## Graphical Abstract

**Graphical Abstract:**
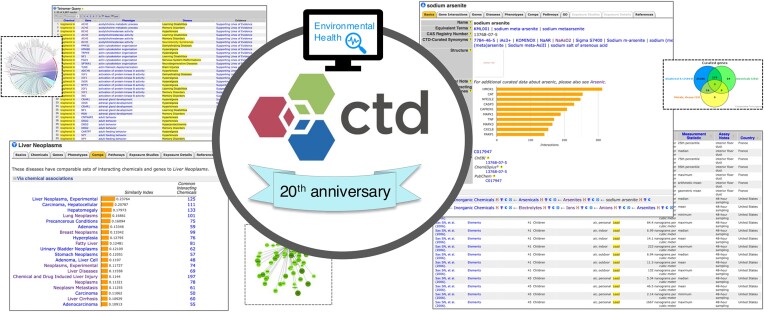

## CTD’s 20th year anniversary

**Figure 1.**
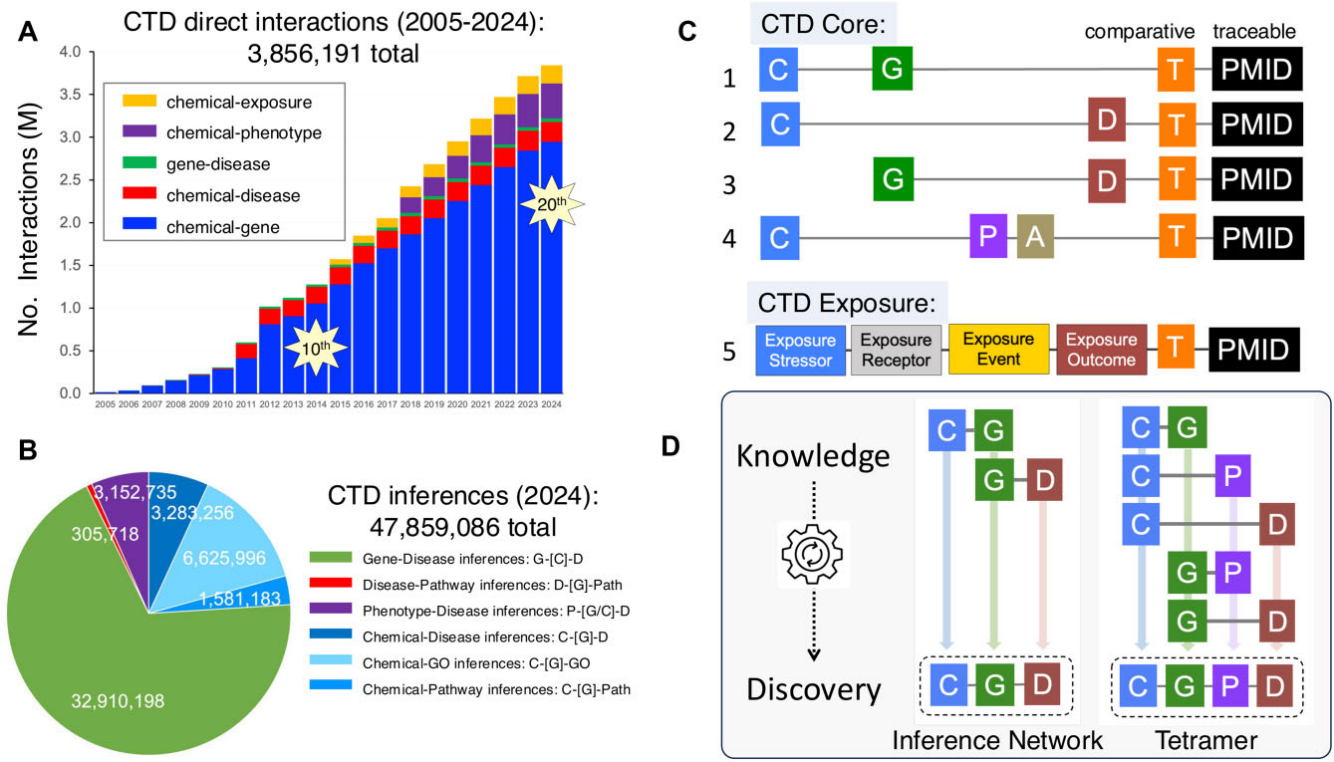
(**A**) Over the last 20 years, CTD has grown to >3.8 million manually curated direct interactions (*y*-axis in millions) and has expanded with new content (chemical-exposure statements in 2015 and chemical–phenotype interactions in 2018). CTD’s 10th and 20th anniversaries are indicated, but counts for year 2024 are incomplete. (**B**) Integration of the 3.8 million direct interactions generates almost 48 million inferences, the majority of which (over 32 million) are for gene–disease inferences via a shared chemical: G–[C]–D. Phenotype–disease inferences can be made via either shared genes or shared chemicals and are reported together, as such: P–[G/C]–D. CTD is updated on a monthly basis and the most current counts are available at https://ctdbase.org/about/dataStatus.go. (**C**) CTD biocurators manually curate five types of interactions from the scientific literature. CTD Core captures four relationships between chemicals (C), genes (G), phenotypes (P), anatomical terms (A) and diseases (D). Interactions are contextualized with taxa (**T**), making the database comparative, and are traceable to source articles by PubMed identifiers (PMID). For simplicity, the 50 predicates and 15 qualifiers are not shown. In CTD Exposure, biocurators construct detailed statements using 20 controlled vocabularies to link 33 distinct data concepts for the four exposure categories relating real-world environmental measurements: exposure stressor, exposure receptor, exposure event and exposure outcome. (**D**) CTD uses data integration to transform curated knowledge into potential discoveries. Chemical–gene and gene–disease curated interactions are combined to generate ‘Inference Networks’ that connect chemicals to diseases by inferred gene intermediates. Similarly, five curated interactions are integrated to compute a ‘Tetramer’ that links a chemical, gene, phenotype and disease in a potential stepwise mechanistic pathway.

The Comparative Toxicogenomics Database (CTD; https://ctdbase.org) was launched 20 years ago (12 November 2004) as a novel ‘prototype’ public resource that imported and integrated data from the scientific literature relating nucleotide/protein sequences, environmental toxicants and taxa to explore toxicogenomic relationships from comparative species (1). Since that humble beginning, CTD has evolved and flourished into a highly cited, robust and sophisticated digital ecosystem. As of August 2024, CTD includes 3.8 million direct interactions manually curated from >149 000 scientific articles (Figure 1A), interrelating >17 700 chemicals, 55 400 genes, 6700 phenotypes, 7200 diseases, 214 000 exposures and 980 anatomical terms for over 630 species. These direct interactions are integrated (see below) to generate an additional 48 million inferred relationships (Figure 1B). Combining these data with imported gene Gene Ontology annotations (2), gene pathways from Kyoto Encyclopedia of Genes and Genomes (KEGG) and Reactome (3,4) and gene–gene interactions from BioGRID (5) results in 94 million toxicogenomic relationships in CTD (https://ctdbase.org/about/dataStatus.go). This vast data landscape helps address knowledge gaps and enables construction of testable mechanistic hypotheses for environmental health. Here, we reflect on the historical uniqueness, complexity and utility of CTD as well as present our most recent updates.

One of CTD’s first milestones was to design a curation paradigm that enabled biocurators to read the literature and manually code interactions using formatted, structured notation and controlled vocabularies and ontologies to capture any type of a chemical-induced biological effect (6,7). In the CTD Core module, biocurators collect four types of direct interactions from scientific articles: chemical–gene/protein interactions, chemical–phenotype interactions, chemical–disease associations and gene–disease associations (Figure 1C). This paradigm is distinct in three important ways. First, CTD does not limit data collection to any single organism, but captures toxicogenomic information for all Eumetazoa. Comparing toxicology data across diverse species was recognized early on as a critical factor to better understand toxicity mechanisms, susceptibility and drug safety (8,9); however, when CTD was launched in 2004, most publicly curated data were siloed in independent single model organism databases, making cross-species comparison cumbersome. CTD addresses that shortcoming by capturing toxicogenomic data for any eumetazoan species encountered in an article. Second, CTD eschews the use of free text, requiring annotations to be constructed using controlled vocabularies and ontologies that align with CTD’s data FAIRness policy (https://ctdbase.org/about/ctdDataFairness.jsp); this ensures that chemical, gene, phenotype, disease, anatomy, interaction and taxa information are standardized across all curated articles, no matter where or when the paper was originally published (e.g. the oldest paper curated in CTD is from 1947). Third, CTD interactions are formatted using a sentence structure (e.g. ‘bisphenol A results in increased cleavage of CASP3 protein in *Mus musculus*’). Here, controlled terms are organized in a structured format to create a chemical-induced biological statement, including a subject (bisphenol A), predicate (increased cleavage) and direct object (CASP3 protein), all contextualized with a taxon (*M. musculus*). This paradigm ensures that cross-species heterogeneous data now become standardized, harmonized, centralized and interoperable, as well as facilitates the emergence of systems toxicology networks (10).

Ten years later (March 2015), CTD increased its complexity, uniqueness and utility by launching another novel module called CTD Exposure (11). This module was designed to help characterize the human exposome, report real-world chemical stressor and biomarker measurements, and address the scientific community’s need to couple the exposome concept to mechanistic toxicology (12). In CTD Exposure, biocurators critically read complex exposure articles and utilize over 20 vocabularies to construct a detailed statement (composed of up to 33 unique data fields) interrelating an exposure stressor, exposure receptor, exposure event and exposure outcome (Figure 1C), which reflect the four main concepts of the exposure ontology (13). This module reports the real-world measurements of environmental toxicants and biomarkers with their associated adverse outcomes, collecting detailed information about the chemical stressor and its source, the receptor demographics (e.g. cohort size, age distribution, gender, smoking status, race and ethnicity), the event particulars (e.g. biomarker levels, assayed medium, statistics, time frame, country, state and project title) and potential outcomes (e.g. phenotypes, diseases and statistical strength). Importantly, we use the same controlled vocabularies (chemical, gene, phenotype, anatomy and disease) for both CTD Core and CTD Exposure curation efforts, enabling seamless integration and complementarity. CTD Core curation (from laboratory experiments) can help inform potential mechanistic pathways for environmental health, and CTD Exposure content (from real-world reporting) informs hypotheses for additional laboratory testing and validation. Together, the two interoperable modules empower users to explore environmental health content from populations to molecules, and vice versa.

CTD’s second milestone was to develop data integration strategies that transform this curated knowledgebase into a toxicogenomic discoverybase (Figure 1D). Because CTD’s manually curated interactions are constructed using controlled vocabularies, the datasets can now be integrated with one another to generate novel discoveries. For example, chemical–gene and gene–disease direct interactions are combined to produce ‘inferences’ following Swanson’s ABC model for knowledge transfer (14): If chemical A interacts with gene B, and gene B is associated with disease C, then chemical A has an inferred relationship to disease C, via gene B (15). In a similar approach, five independently curated statements from CTD can be integrated to generate a ‘CGPD tetramer’: a mechanistic construct that links an initiating chemical with an intermediate gene to modulate a key biological phenotype that can play a role in a disease (16). These inferred and computationally derived relationships provide users with potential molecular mechanisms that can be assembled to construct chemical–disease pathways by filling the knowledge gaps between an exposure and outcome. Thus, CTD functions as both a knowledgebase (directly curated interactions from the literature) and a discoverybase (integration-derived predictions that can potentially fill knowledge gaps).

Paralleling this increased growth in curated content, CTD’s software engineering, technological perspectives and infrastructure have also grown significantly over the last 20 years (Table 1). We have made considerable investments and upgrades in hardware and software development, with a more robust software engineering team.

**Table 1.** CTD computing upgrades from 2004 to 2024

Description	2004	2024	Fold increase (as applicable)
Operating system	Solaris 9	Rocky Linux 8.9	
Database management system	Oracle 10	PostgreSQL 14	
CPU cores	1	32	32
CPU clock	750 MHz	3.6 GHz	4.8
RAM	2 GB	768 GB	384
Storage (total)	432 GB	45 TB	107
Computer room (CR)	None (basement)	NCSU Eastern Data Center	
CR HVAC	None	Full	
CR fire suppression	None	Full	
CR wildlife	Spiders, snakes	None (that we know of)	

To aid users in exploring the CTD landscape, we provide a comprehensive suite of search menus (https://ctdbase.org/search/), analytical tools (https://ctdbase.org/tools/) and downloadable files (https://ctdbase.org/downloads/). Additionally, we have published 50 peer-reviewed publications (https://ctdbase.org/about/publications/#ctdpubs) and presented at 78 national and international conferences (https://ctdbase.org/about/publications/#ctdpres) to promote CTD’s curation paradigms, tools, features, data content and functionalities, with numerous case studies to demonstrate how users can employ CTD to ask, address and answer scientific questions related to environmental health.

Over the last two decades, CTD has evolved into an authoritative ‘golden set database’ (17), acting as a comprehensive resource for the scientific community. Currently, CTD has over 8000 cumulative Google Scholar citations and a citation rate of three per day since 2023. Furthermore, 227 external databases link to or utilize CTD information (https://ctdbase.org/about/publications/#use), helping to further disseminate CTD data.

## New features

### Curation updates: natural language processing and new Exposure Curation Tool

CTD curation is limited to scientific publications indexed in PubMed (https://pubmed.ncbi.nlm.nih.gov/). To prioritize articles for curation, we use a complementary, two-pronged process (chemical-centric and targeted journals) that promotes data completeness and increases data currency, as previously described in detail (6,18,19). For a large corpus of selected articles, we also leverage our text-mining algorithm to score and rank the articles for curation, which we have shown increases both productivity and efficiency (19).

**Figure 2.**
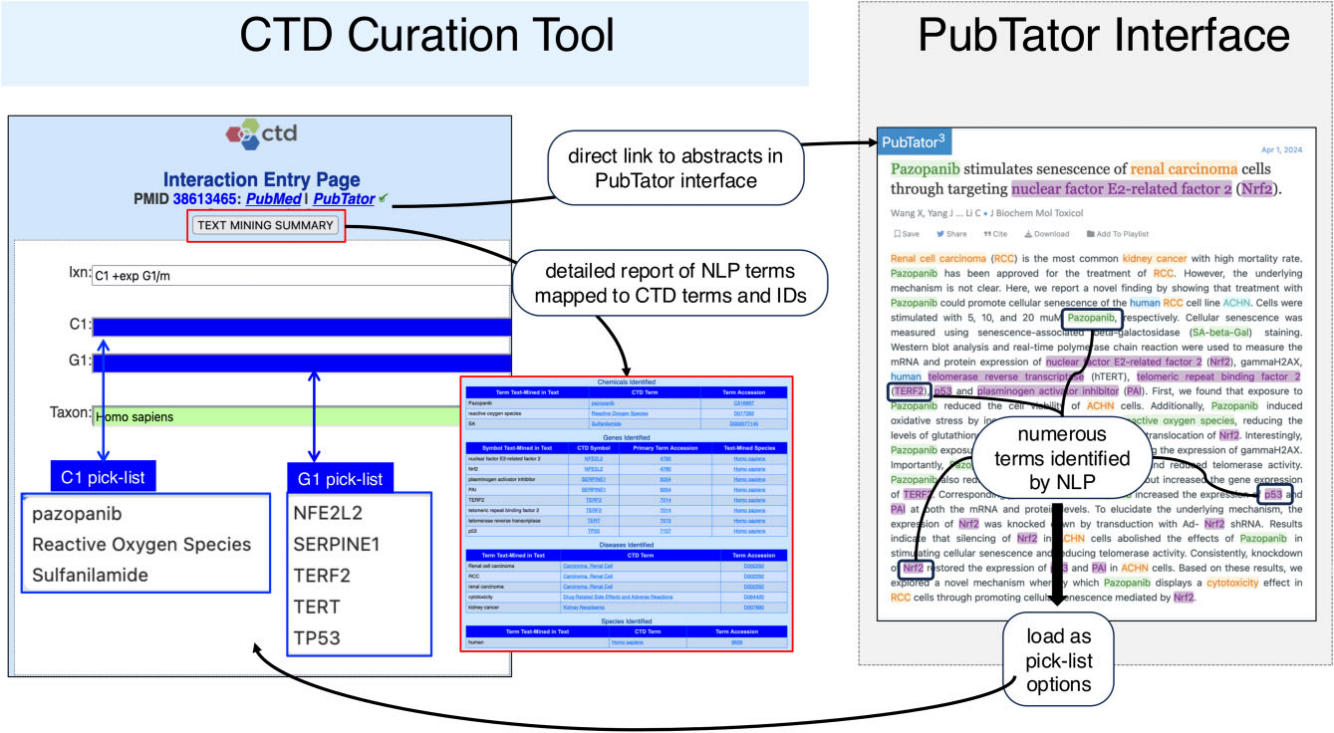
From the CTD Curation Tool, biocurators link to abstracts in the PubTator interface that display the color-coded terms for chemicals, genes, diseases and taxa identified by NLP. A detailed text-mining summary report shows the NLP terms mapped to their corresponding CTD controlled vocabularies and accession identifiers. Additionally, the CTD terms are loaded as pick-list options in the CTD Curation Tool [C1, chemicals; G1, genes; D1, diseases (not shown)] for biocurators to select when entering interactions in the tool, streamlining the curation process.

To further enhance and streamline curation, as well as to extend our use of text mining (20), we have now fully incorporated the natural language processing (NLP) capabilities provided by PubTator (21). CTD biocurators access scientific articles using the PubTator interface, where abstracts are illuminated with color-coded terms for chemicals, genes, diseases and species by PubTator’s NLP (Figure 2). Additionally, these NLP-identified terms are summarized as a report and imported directly into the CTD Curation Tool data entry fields for biocurators to select using a pick-list when curating (Figure 2).

The CTD Exposure module continues to expand and now contains 214 000 exposure statements relating 1500 exposure stressors, 1400 human genes and 1000 exposure outcomes (500 phenotypes and 500 environmental diseases), manually curated from over 3400 exposure articles (https://ctdbase.org/about/dataStatus.go). This module requires its own data collection platform, distinct from the CTD Core Curation Tool that has been operational since 2010 (6). We have now implemented a new CTD Exposure Curation Tool (ECT), which enables biocurators to directly submit their curated annotations derived from exposure articles (11). The new online ECT ensures both high-quality content and consistency as it automatically performs dozens of quality control checks in real time to validate data entry, enabling biocurators to immediately fix any detected error or conflicting value at the point of curation.

### Tool updates: CTD tetramers and pathway view

**Figure 3.**
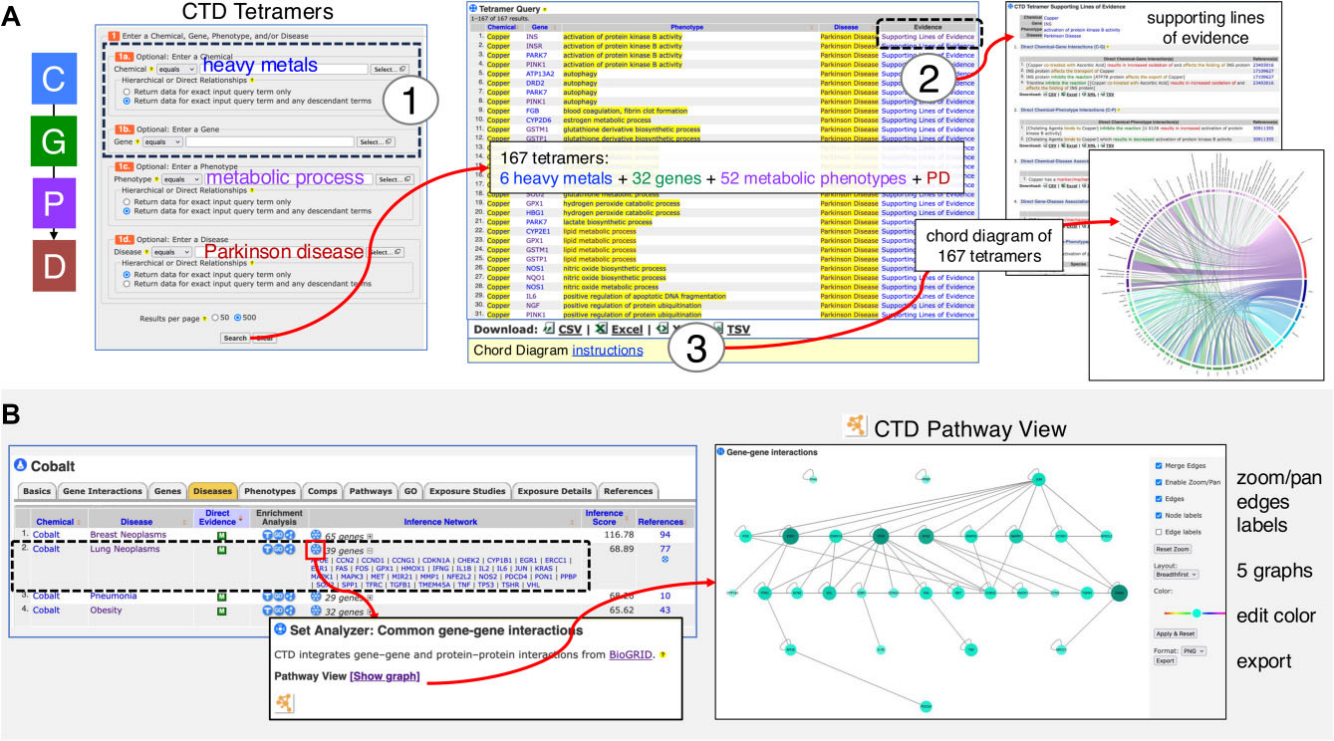
(**A**) Three enhancements have been added to the CTD Tetramers tool (https://ctdbase.org/query.go?type=tetramer) that is used to generate CGPD tetramers for constructing chemical–disease mechanistic pathways. Now users can include a chemical and/or gene (dotted black box), in addition to a phenotype and disease, to the query, enabling focused searches for specific molecular events (circle 1). Here, a multi-parameter query searches for CGPD–tetramers that can connect ‘heavy metals’ (chemical) to ‘Parkinson disease’ (disease) via an intermediate ‘metabolic process’ phenotype. Optional filters are selected to retrieve hierarchical descendant terms for both the chemical and phenotype terms. This query retrieves 167 CGPD–tetramers, composed of 6 heavy metals, 32 genes and 52 metabolic process phenotypes linked to Parkinson disease. A new ‘Evidence’ column (circle 2) provides the five supporting lines of evidence (and their source articles) used to construct the tetramer. The tabular output can be transformed into a chord diagram following a set of user-friendly instructions (circle 3); chord diagrams help visualize key mechanisms. (**B**) Gene–gene interaction maps can be easily generated from CTD ‘Gene Inference Networks’. Here, the metal cobalt is inferred to lung neoplasms via 39 genes (black dotted box). Clicking the tool icon (in front of the gene list) sends the genes to the Set Analyzer tool and constructs an interaction map (based upon BioGRID data), which can be seen by clicking the Pathway View link. The tool has been updated with new customizable features, including the ability to adjust edge and label displays, zoom/pan, change node colors, select from five graph layouts and export maps. Alternatively, users can input their own gene list using the Set Analyzer tool directly (https://ctdbase.org/tools/analyzer.go) to generate any gene–gene interaction map.

CGPD tetramers (Figure 1D) provide insight into potential molecular mechanisms and can be assembled to construct complex chemical-induced pathways that have been used to help fill knowledge gaps and design adverse outcome pathways (AOPs) for a diversity of environmental health studies (22–26). In 2022, we released the online tool *CTD Tetramers* that empowers users to generate tetramers for any phenotype or disease of interest (16). We have now enhanced this tool (https://ctdbase.org/query.go?type=tetramer) in three significant ways. First, users now can include chemicals and/or genes in their tetramer queries, in addition to phenotypes or diseases. This allows users to search for targeted datasets for any of the four components of a tetramer (i.e. chemical, gene, phenotype and/or disease), or a combination of components, such as how heavy metals can be linked to Parkinson disease via a metabolism-related phenotype (Figure 3A). This enhancement provides greater querying power to discover specific sets of tetramers that can be used to help construct and refine chemical-induced pathways and AOPs. Second, we now include an ‘Evidence’ column for each tetramer that shows the five supporting lines of evidence (and their source articles) used to generate the tetramer (Figure 3A). This transparency allows users to evaluate the strength of the evidence underlying each tetramer. Third, we provide a method to transform tabular tetramer query results into a novel chord diagram to help visualize and identify potential key molecular mechanisms (27). Chord diagrams provide an elegant visual representation of the tetramers connecting a chemical exposure to an adverse outcome, which can be used to understand the possibe underlying mechanisms and guide AOP models.

Additionally, we have updated CTD’s Pathway View functionality, which uses web-based Cytoscape (28) for visualizing gene–gene networks for gene sets computed as either CTD ‘Gene Inference Networks’ (Figure 3B) or input directly using the CTD Set Analyzer tool (https://ctdbase.org/tools/analyzer.go). The modernized Pathway View maps are customizable with five new preset graphing layouts. In addition, users can drag nodes, remove node and edge labels, change node colors, zoom and pan, and export maps in two formats (PNG or JPG). This new functionality leverages the JavaScript implementation of the Cytoscape library, which enables layout management, viewport adjustments and other graph operations. To support additional physics simulation layouts with animation, two extension libraries have also been integrated into CTD’s Jakarta EE-based JSP/Servlet architecture.

### Other enhancements: API and infrastructure upgrades

We improved the visibility and functionality of CTD’s application programming interfaces (APIs) to address the increased interest from external resources to retrieve CTD content computationally. An explicit link to our API is now found in the base menu options under the ‘Analyze’ tab of every CTD web page (https://ctdbase.org/help/linking.jsp#batchqueries). As well, the API has been updated to allow extraction of both phenotype-based curation and phenotype–disease inferences.

To address the increased content within CTD and provide the computational capacity necessary to support future needs, significant upgrades were made to CTD’s infrastructure. Existing computer hardware was replaced with three new servers that have dual 3.6 GHz 16-core processors and 768 GB memory capacity (Table 1). We also migrated to a new 45 TB NetApp all-flash (i.e. solid-state drive) storage array. These upgrades coincided with a physical move to NCSU’s new state-of-the-art facility Eastern Data Center with its much-improved 25 Gb/s network. In this updated infrastructure, we also migrated to new server operating systems, PostgreSQL database management systems, Tomcat application servers, and the latest versions of third-party libraries and software.

## Summary

1. Over the last 20 years, CTD has evolved into a premier toxicogenomic digital ecosystem, with new curation modules, features and tools to support the needs of the scientific community.
2. Manually curated CTD content increased by 13% to >3.8 million direct interactions since our last update; when integrated with other data types, CTD now generates over 94 million toxicogenomic relationships (https://ctdbase.org/about/dataStatus.go).
3. Terms identified by NLP from PubTator are imported into the CTD Curation Tool to enhance curation processes.
4. A new online ECT is now used in our curation process to increase efficiency and accuracy.
5. CTD Tetramers tool was enhanced with features allowing for more sophisticated querying, increased transparency and traceability, and the ability to visualize results as chord diagrams.
6. Pathway View tool used to map gene networks was updated with new functionalities.
7. Visibility and function of CTD’s API data retrieval system were increased.
8. Significant upgrades and investments to CTD software engineering and technical infrastructure are reported.

## Data Availability

CTD is publicly available at https://ctdbase.org. All aspects of CTD are freely available to non-commercial users; commercial users are asked to purchase a license in order to download CTD data files to their systems. To cite CTD, please see https://ctdbase.org/about/publications/#citing. If interested in establishing links to CTD data, please notify us (https://ctdbase.org/help/contact.go) and follow the instructions at https://ctdbase.org/help/linking.jsp. External resources using CTD content are promoted at https://ctdbase.org/about/publications/#use.
