# Correction to ‘Comparative Toxicogenomics Database's 20th anniversary: update 2025’

**DOI:** 10.1093/nar/gkae1185

**Published:** 2024-11-22

This is a correction to: Allan Peter Davis, Thomas C Wiegers, Daniela Sciaky, Fern Barkalow, Melissa Strong, Brent Wyatt, Jolene Wiegers, Roy McMorran, Sakib Abrar, Carolyn J Mattingly, Comparative Toxicogenomics Database's 20th anniversary: update 2025, *Nucleic Acids Research*, 2024, gkae883, https://doi.org/10.1093/nar/gkae883

In the originally published version of this article, ‘Comparative Toxicogenomics Database’ was not correctly capitalized in the article title. The publisher apologizes for the error.

